# Supplementary material for: Inspired by the Nature: A Post-printed Strategy to Efficiently Elaborate Parahydrophobic Surfaces
Source: Biomimetics (Basel). 2022 Aug 28;7(3):122. doi: 10.3390/biomimetics7030122 (PMC9496598; doi:10.3390/biomimetics7030122)
Supplement: Supplementary file 1 [file biomimetics-07-00122-s001.zip › biomimetics-1784356-supplementary.pdf]

Supplementary Materials: Inspired by the Nature: A Post-printed Strategy to Efficiently Elaborate Parahydrophobic Surfaces

Jordy Queiros Campos [1], Caroline R. Szczepanski [2], Marie Gabrielle Medici [1] and Guilhem Godeau [1,3,*]

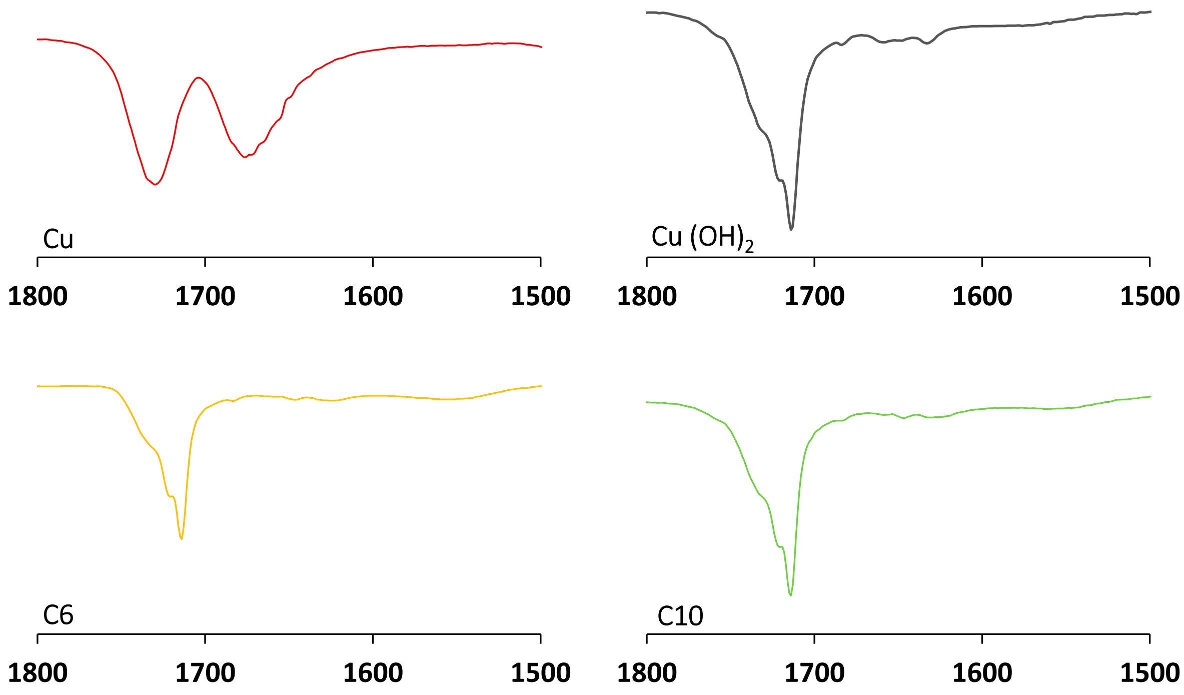

Figure S1. Examples of IR spectra for raw and functionalized surfaces.
